# Regulation of iNOS function and cellular redox state by macrophage *Gch1* reveals specific requirements for tetrahydrobiopterin in NRF2 activation

**DOI:** 10.1016/j.freeradbiomed.2014.10.575

**Published:** 2015-02

**Authors:** Eileen McNeill, Mark J. Crabtree, Natasha Sahgal, Jyoti Patel, Surawee Chuaiphichai, Asif J. Iqbal, Ashley B. Hale, David R. Greaves, Keith M. Channon

**Affiliations:** a Division of Cardiovascular Medicine, British Heart Foundation Centre of Research Excellence, John Radcliffe Hospital, University of Oxford, Oxford, UK; b Wellcome Trust Centre for Human Genetics, University of Oxford, Oxford, UK; c Sir William Dunn School of Pathology University of Oxford, Oxford, UK

**Keywords:** As3MT, arsenic(III)-methyltransferase, BH4, tetrahydrobiopterin, [Fe(DETC)_2_], colloid iron (II) diethyldithiocarbamate, GTPCH, GTP cyclohydrolase 1, 2-HE, 2-hydroxyethidium, iNOS, inducible nitric oxide synthase, ROS, reactive oxygen species., Tetrahydrobiopterin, BH4, GCH1, GTPCH, Macrophage, NOS2, iNOS, Nitric oxide, As3MT

## Abstract

Inducible nitric oxide synthase (iNOS) is a key enzyme in the macrophage inflammatory response, which is the source of nitric oxide (NO) that is potently induced in response to proinflammatory stimuli. However, the specific role of NO production, as distinct from iNOS induction, in macrophage inflammatory responses remains unproven. We have generated a novel mouse model with conditional deletion of *Gch1*, encoding GTP cyclohydrolase 1 (GTPCH), an essential enzyme in the biosynthesis of tetrahydrobiopterin (BH4) that is a required cofactor for iNOS NO production. Mice with a floxed *Gch1* allele (*Gch1*^fl/fl^) were crossed with Tie2cre transgenic mice, causing *Gch1* deletion in leukocytes (*Gch1*^fl/fl^Tie2cre). Macrophages from *Gch1*^fl/fl^Tie2cre mice lacked GTPCH protein and de novo biopterin biosynthesis. When activated with LPS and IFNγ, macrophages from *Gch1*^fl/fl^Tie2cre mice induced iNOS protein in a manner indistinguishable from wild-type controls, but produced no detectable NO, as judged by L-citrulline production, EPR spin trapping of NO, and by nitrite accumulation. Incubation of *Gch1*^fl/fl^Tie2cre macrophages with dihydroethidium revealed significantly increased production of superoxide in the presence of iNOS expression, and an iNOS-independent, BH4-dependent increase in other ROS species. Normal BH4 levels, nitric oxide production, and cellular redox state were restored by sepiapterin, a precursor of BH4 production by the salvage pathway, demonstrating that the effects of BH4 deficiency were reversible. *Gch1*^fl/fl^Tie2cre macrophages showed only minor alterations in cytokine production and normal cell migration, and minimal changes in basal gene expression. However, gene expression analysis after iNOS induction identified 78 genes that were altered between wild-type and *Gch1*^fl/fl^Tie2cre macrophages. Pathway analysis identified decreased NRF2 activation, with reduced induction of archetypal NRF2 genes (*gclm*, *prdx1*, *gsta3*, *nqo1*, and *catalase*) in BH4-deficient *Gch1*^fl/fl^Tie2cre macrophages. These findings identify BH4-dependent iNOS regulation and NO generation as specific requirements for NRF2-dependent responses in macrophage inflammatory activation.

## Highlights

• *Gch1*^*−/−*^ macrophages lack GTPCH protein expression, rendering cells BH4 deficient.
• *Gch1*^*−/−*^ cells express normal iNOS protein levels, but lack nitric oxide production.
• BH4-deficient cells exhibit higher cellular ROS production.
• Alterations in NO and ROS production can be reversed by pharmacological BH4 rescue.
• Stimulated BH4-deficient cells show defective NRF2-dependent gene expression.

## Graphical abstract

**Figure:**
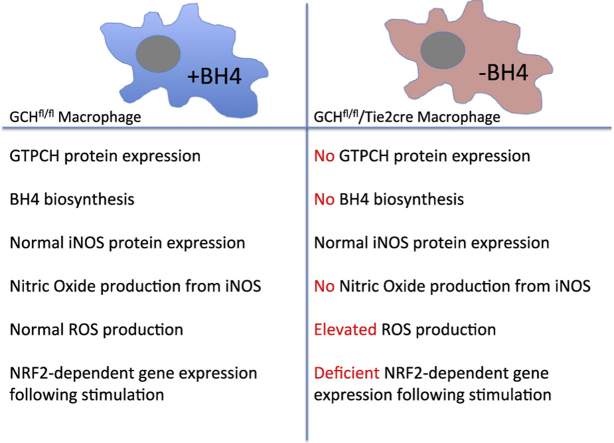

## Introduction

Macrophages are pivotal cells of the innate immune system, and are a source of both pro- and anti-inflammatory cytokines, capable of phagocytosing apoptic cells and have a potent antimicrobial arsenal [1]. Archetypal proinflammatory macrophages are activated by interferon-γ and microbial products such as LPS, leading to production of proinflammatory cytokines and high levels of nitric oxide (NO), due to the induction of nitric oxide synthase. Generation of NO by inducible nitric oxide synthase (iNOS, encoded by *NOS2*) is implicated in critical functions such as microbial killing and immune regulation [2]. However, inflammatory cells including macrophages generate high levels of reactive oxygen specifies (ROS), that also play key roles in host defense and immunity. Interactions between NO and ROS in cellular redox signaling are important determinants of the inflammatory response, through effects on redox-sensitive gene expression. For example, the activation of NRF2, leading to induction of antioxidant defense genes, is stimulated by NO through nitrosylation of KEAP1 [3], but is also regulated by ROS [4] and other oxidatively modified molecules [5]. Indeed, incomplete understanding of the balance among NO, ROS, and downstream redox effects may underlie the inconsistent effects of “antioxidant” therapies [6].

The evidence that important redox functions for iNOS in macrophage activation are mediated by NO comes from analysis of mice with genetic deletion of *Nos2*, which have altered systemic inflammatory responses to systemic infection, resulting in partial protection from septic shock [2,7]. The effects of *Nos2* deletion are presumed to result from loss of NO generation by iNOS. However, deletion of *Nos2* results in total absence of iNOS expression and activity, and this is not synonymous with selective loss of NO generation, because the NOS enzymes are complex oxido-reductases with the potential to produce ROS as well as, or instead of, NO [8]. Specifically, the NOS enzymes require the pterin cofactor tetrahydrobiopterin (BH4) for NO production [9]. BH4 participates in electron transfer during the two-step oxidation of L-arginine to L-citrulline via an *N-*hydroxyarginine intermediate. In the absence of BH4, NOS enzymes are unable to generate NO by oxidation of L-arginine, and become “uncoupled,” whereby reduction of molecular oxygen, driven by electron flow from the NOS reductase domain, can generate superoxide or other ROS. Uncoupled NOS appears to play important roles in pathophysiologic regulation of ROS in, for example, cardiovascular disease [10]. Inhibition of iNOS function by BH4 structural analogues *in vitro* can block iNOS-mediated NO production, and may have effects on the systemic response to septic shock [11,12]. However, the physiological requirement for BH4 in iNOS function is unclear. In particular, it is not known whether the role of BH4 in inflammatory cell function alters the response to cytokine stimulation, or the downstream consequences of iNOS signaling mediated by either NO or ROS. These questions are important because understanding the mechanisms regulating iNOS signaling in inflammation, via either NO or ROS, is critical in designing rational therapeutic approaches to target iNOS function in conditions such as host defense, immune function, and sepsis.

We hypothesized that deletion of *Gch1* in macrophages would prevent NO production by iNOS and allow dissection of putative roles for iNOS-derived NO, vs other functions of iNOS, such as ROS production, that are not dependent on NO production. We generated a novel mouse model with targeted deletion of *Gch1*, the enzyme required for BH4 synthesis, in order to prevent NO production by iNOS, but maintain ROS production. We investigated the effects of “NO-dead” iNOS on macrophage activation and redox-dependent gene expression.

## Materials and methods

### Animals

All animal studies were conducted with ethical approval from the Local Ethical Review Committee and in accordance with the UK Home Office regulations (Guidance on the Operation of Animals, Scientific Procedures Act, 1986). Mice were housed in ventilated cages with a 12-h light/dark cycle and controlled temperature (20–22 °C), and fed normal chow and water ad libitum.

### Generation of Gch1 knockout mice

**Fig. 1:**
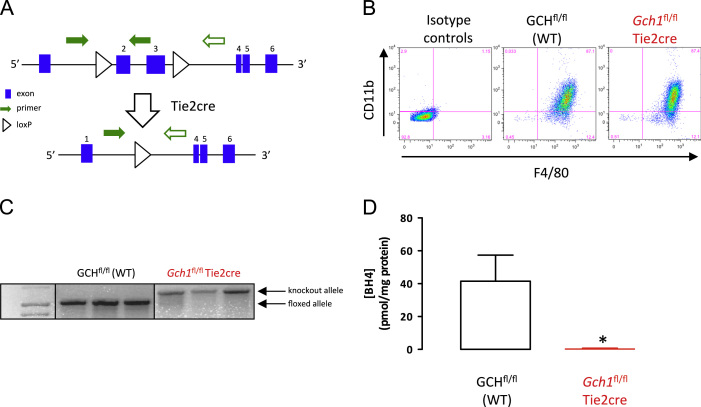
(A) Schematic showing the targeting of the mouse *Gch1* locus indicating the position of the loxP sites flanking exons 2 and 3, which encode the active site of the GTPCH enzyme. Arrows indicate the position of primers that produce the 1030-bp product from the floxed allele (solid green arrows) and the 1392-bp product following excision of the floxed DNA (solid and open green arrows). (B) Flow cytometry dot plots show expression of CD11b and F4/80 on the cell surface of wild-type (WT) and *Gch1*^fl/fl^Tie2cre bone marrow-derived macrophages, indicating similar differentiation in both genotypes after 7 days of culture of bone marrow cells in macrophage differentiation media. (C) Genomic PCR shows the presence of the floxed allele in WT cells and the near complete excision of the floxed allele in *Gch1*^fl/fl^Tie2cre bone marrow-derived macrophages. (D) Measurement of BH4 in the cells shows a near complete loss of BH4 in *Gch1*^fl/fl^Tie2cre cells. (*n*=3, *P*<0.05).

A novel mouse model of BH4 deficiency was created by conditional deletion of *Gch1,* that encodes GTP cyclohydrolase I the rate limiting enzyme in BH4 biosynthesis [13]. Exons 2 and 3 of *Gch1*, encoding for the active site of GTPCH, were flanked by loxP sites in a targeting construct that was used to produce *Gch1*^*fl/fl*^ mice following homologous recombination in ES cells (Fig. 1A). These mice were crossed with Tie2cre transgenic mice to produce *Gch1*^*fl/fl*^Tie2cre mice where *Gch1* is deleted in endothelial and bone marrow-derived cells [14–16]. The Tie2cre transgene is active in the female germline; hence only male animals are used to establish breeding pairs to maintain conditional expression. Mice were genotyped using primers targeted against the floxed *Gch1* allele, and in a separate reaction for the presence of the cre sequence. Experiments were performed using bone marrow isolated from age and sex matched littermate animals. Animals were genotyped using DNA prepared from ear notches using the following PCR primers: Cre, 5′ GCATAACCAGTGAAACAGCATTGCTG 3′ and 5′ GGACATGTTCAGGGATCGCCAGGCG 3′; *Gch1* floxed allele, 5′GTCCTTGGTCTCAGTAAACTTGCCAGG3′ and 5′GCCCAGCCAAGGATAGATGCAG3′.

### Isolation of murine bone marrow-derived macrophages

Bone marrow was obtained by flushing the femur and tibia of adult mice with PBS. A single cell suspension was prepared by passing the bone marrow through a 70 µm cell strainer. Cells were then cultured in 10 cm nontissue culture treated dishes for 7 days in DMEM:F12 (Invitrogen) supplemented with 100 U/ml penicillin and 100 ng/ml streptomycin (Sigma), 10% (v/v) fetal bovine serum (PAA Laboratories), 5 mM L-glutamine (Sigma), and 10–15% (v/v) L929 conditioned medium at 37 °C and 5% CO_2_. The differentiation of the cells was confirmed using flow cytometry using a CyAn ADP (Beckton Coulter) for data acquisition and Flow Jo (TreeStar Inc.) for analysis. Macrophages were defined as being CD11b (PerCP conjugated) and F4:80 (APC conjugated, both Biolegend) positive cells, as judged against isotype controls conjugated with the same fluorochromes (Biolegend).

### Stimulation of bone marrow-derived macrophages

Following differentiation cells were harvested and plated into 6- or 12-well plates containing serum-free media (Optimem supplemented with 100 U/ml penicillin and 100 ng/ml streptomycin and 0.2% (w/v) low-endotoxin bovine serum albumin (Sigma)). Cells were stimulated with 10 ng/ml IFNγ (Peprotech EC) and 100 ng/ml LPS (Sigma) for 24 h, parallel wells were left unstimulated. After 24 h cell pellets, and cell culture supernatants were collected, or the cells subjected to biochemical analysis.

### Genomic DNA production and excision PCR

Genomic DNA for detection of the excised allele was produced using the Qiamp kit (Qiagen). The floxed and excised allele were detected using the following primers: 5′GTCCTTGGTCTCAGTAAACTTGCCAGG3′, 5′GCCCAGCCAAGGATAGATGCAG3′, and 5′GCTCATCCCCCACACTTGTCTT3′. The floxed allele yields a 1030-bp product and the excised allele a product of 1300 bp.

### Determination of tetrahydrobiopterin levels

BH4 and oxidized biopterins (BH2 and biopterin) were determined by high-performance liquid chromatography (HPLC) followed by electrochemical and fluorescent detection, respectively, following an established protocol [17]. Cell pellets were freeze–thawed in ice-cold resuspension buffer (50 mM phosphate-buffered saline, 1 mM dithioerythriol, 1 mM EDTA, pH 7.4). After centrifugation at 13,200 rpm for 10 min at 4 °C, supernatant was removed and ice-cold acid precipitation buffer (1 M phosphoric acid, 2 M trichloroacetic acid, 1 mM dithioerythritol) was added. Following centrifugation at 13,200 rpm for 10 min at 4 °C, the supernatant was removed and injected onto the HPLC system. Quantification of BH4 and oxidized biopterins was obtained by comparison with external standards and normalized to protein concentration, determined by the BCA protein assay (Pierce).

### Nitrite determination

Nitrite accumulation was measured in samples of cell culture medium using the Griess assay with colorimetric detection in 96-well plates, as described [18].

### Determination of NO production by electron paramagnetic resonance

Electron paramagnetic resonance (EPR) spectroscopy was used to quantify aortic NO production, as described previously [19]. Briefly, cultured macrophages were incubated with colloid iron (II) diethyldithiocarbamate [Fe(DETC)_2_] (285 µmol/L) at 37 °C for 90 min. After incubation, cells were harvested and snap-frozen in a column of Krebs-Hepes buffer in liquid nitrogen, and EPR spectra were obtained using an X-band EPR spectrometer (Miniscope MS 200; Magnettech). Signals were quantified by measuring the total amplitude, after correction of baseline, and after subtracting background signals from incubation with colloid Fe(DETC)_2_ alone.

### Western blot analysis

Western blot analysis was performed using antibodies against murine GTPCH (a gift from S. Gross), iNOS (BD Pharmigen), and β-tubulin (Abcam), using standard protocols. iNOS monomer and dimer analysis was conducted on nonreduced samples with all elements of the experiment conducted at 4 °C.

### Analysis of NO synthesis by iNOS

NO synthesis by iNOS was assessed by conversion of [^14^C]L-arginine to citrulline, in the presence and absence of *N-*methyl-L-arginine, as described previously [20]. Briefly, macrophages were incubated for 2 h at 37 °C in 200 μl Krebs-Hepes buffer containing [^14^C]arginine (2 μl of 50 μCi/mL, Hartmann Radiochemicals). Samples were run on a SCX 300 cation-exchange HPLC column (Sigma) with online scintillation detection. Background signals were corrected from samples incubated with [^14^C]arginine alone, without cells.

### Quantification of intracellular superoxide production by dihydroethidine HPLC

Superoxide production was quantified by measuring production of 2-hydroxyethidium from dihydroethidium using HPLC [21,22]. Cultured macrophages were pretreated with or without 2 mM L-NAME (Sigma), 100 U/ml PEG-SOD (Sigma), or 5 µM Sepiapterin (Schircks) prior to addition 25 µM DHE (Invitrogen) for 30 min before being harvested for separation of DHE using a gradient HPLC system (Jasco, UK) with an ODS3 reverse phase column (250 mm, 4.5 mm, Hichrom UK) and quantified using a fluorescence detector set at 510 nm (excitation) and 595 nm (emission). To assess oxidative burst, cells were stimulated with 2 μM PMA (Sigma) directly after addition of DHE to the cell culture media.

### Cytokine protein array

Media from stimulated and unstimulated macrophages were harvested after 24 h and any cell debris was removed by centrifugation. The resulting supernatants were applied to Proteome Profiler Arrays (R&D Systems) as a batch. The arrays were developed according to the manufacturer׳s instructions. The intensity of the cytokine signals was quantified from high resolution scans using ImageJ software.

### xCELLigence real-time chemotaxis assay

Bone marrow-derived macrophages were de-adhered following differentiation using PBS/5 mM EDTA and then subjected to chemotaxis assays in CIM 16-well plates using an xCELLigence RTCA-DP instrument as described previously [23]. Briefly, agonists were loaded into the lower chamber of the CIM 16 plate and the upper chamber was attached. The electrode-embedded membrane was equilibrated with prewarmed buffer for 30 min prior to addition of 5×10^4^ cells. Migration was assessed every 5 s for 4 h in total. Data were normalized to wells that received media alone and analysis of area maximum–minimum signal was carried out with RCTA Software version 1.2.1. Data are displayed as cell index relative to media alone control.

### Gene array analysis

Total RNA was extracted from each sample using a mirVana kit (Ambion). The RNA quantity and quality were determined using the Agilent BioAnalyzer 2100 (Agilent Technologies). Gene expression data were obtained by hybridizing a total of 24 mouse samples from two experimental groups, over 4 time points: WT and *Gch1*^fl/fl^Tie2cre at 0, 2, 8, or 24 h post stimulation (*n*=3 per group) to Illumina MouseWG-6(V2) BeadChips. Chips were scanned with Illumina BeadArray Reader; GenomeStudio V2010.1 (Illumina Inc) was used for data extraction. Data underwent variance stabilization and normalization (VSN) algorithm using Limma [24]. Normalized data were imported to Bioconductor (R version 2.14) (Biobase) and comparison by genotype was performed for each time point to identify significantly differentially expressed genes passing a false discovery rate of <0.05 [25]. The lists of differentially expressed genes were then subject to IPA pathway analysis (Ingenuity Systems, http://www.ingenuity.com).

### Quantitative real-time RT-PCR

RNA, prepared as described above, were reverse-transcribed using Superscript II (Life Technologies) and random hexamer primers (Invitrogen) according to standard protocols. A 50 ng RNA equivalent cDNA was used to perform real-time PCR using predesigned TaqMan gene expression assays (Life Technologies) using a BioRad CFX1000. Gene expression data were normalized to β-actin expression.

### Statistical analysis

Data are expressed as mean ± SEM. Comparisons between WT and *GCH1*^*fl/fl*^Tie2cre were made by the unpaired *Student t* test. Experiments testing multiple doses between genotypes were compared by two-way ANOVA, with post tests applied to test for significance between genotypes or treatments as outlined in the figure legends. A *P* value of less than 0.05 was considered statistically significant.

## Results

### Cell-targeted Gch1 deletion reveals a requirement for BH4 biosynthesis in macrophage iNOS function

We generated matched litters of *Gch1*^*fl/fl*^Tie2cre and *Gch1*^*fl/fl*^ mice (hereafter referred to as wild-type) by crossing male *Gch1*^*fl/fl*^Tie2cre and female *Gch1*^*fl/fl*^ mice (Fig. 1A). Bone marrow-derived macrophages were cultured from primary bone marrow, confirmed by identification of the cells as CD11b+ F4/80+ by flow cytometry (Fig. 1B). Genomic PCR of macrophage DNA demonstrated near total excision of the floxed *Gch1* allele in *Gch1*^*fl/fl*^Tie2cre mice (Fig. 1C). Deletion of *Gch1* resulted in a near complete loss of cellular BH4 in bone marrow-derived macrophages from *Gch1*^*fl/fl*^Tie2cre mice compared to wild-type controls (*P*<0.01, Fig. 1D).

**Fig. 2:**
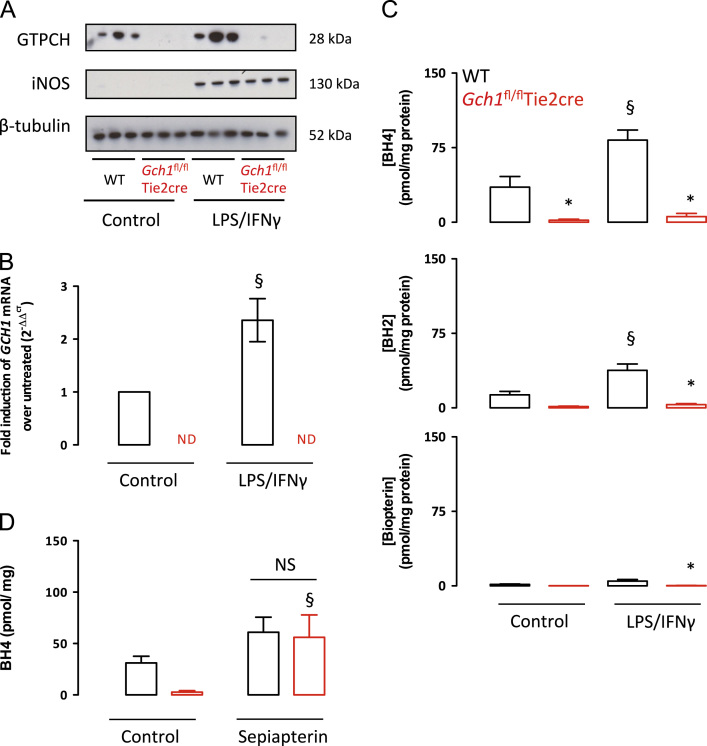
(A) Western blotting shows a lack of expression of GTPCH protein in *Gch1*^fl/fl^Tie2cre macrophages, compared to wild-type (WT) macrophages that show increased expression following activation with LPS (100 ng/ml) and IFNγ (10 ng/ml) for 24 h. Both genotypes of cells showed similar induction of iNOS protein following activation. Equal protein loading was demonstrated by detection of β-tubulin, *n*=3 independent cell preparations. (B) Gene expression analysis of cells 24 h after stimulation showed a significant induction of *Gch1* RNA; in contrast *Gch1* RNA was undetectable in *Gch1*^fl/fl^Tie2cre cells (*n*=3, *P*<0.05). (C) BH4 and BH2 were upregulated in wild-type macrophages following activation. All forms of biopterin were significantly reduced to near undetectable levels in *Gch1*^fl/fl^Tie2cre cells. (D) Treatment of macrophages with 5 μM Sepiapterin for 24 h significantly increased BH4 content in *Gch1*^fl/fl^Tie2cre cells, to levels that were not significantly different from wild-type cells (*n*=4, *P*<0.05). *P<0.05, difference by genotype; § *P*<0.05, difference from control. Two-way ANOVA.

We next tested the effect of cytokine stimulation on iNOS expression and BH4 synthesis in bone marrow-derived macrophages. Stimulation of macrophages with LPS and interferon-γ resulted in marked induction of iNOS protein levels, identified by Western blot, from undetectable levels at baseline, that was not different in *Gch1*^*fl/fl*^Tie2cre compared with wild-type mice (Fig. 2A). In wild-type mice, GTPCH protein was present at baseline and was upregulated in response to LPS and interferon-γ stimulation. However, in *Gch1*^*fl/fl*^Tie2cre mice, no GTPCH protein was detectable either at baseline or after LPS and interferon-γ stimulation. These changes in GTPCH protein were paralleled by quantification of *Gch1* mRNA by RT-PCR, showing an upregulation of *Gch1* expression in wild-type cells after LPS and interferon-γ stimulation, but undetectable *Gch1* expression in either basal or stimulated *Gch1*^*fl/fl*^Tie2cre macrophages (Fig. 2B). Strikingly, levels of BH4, and the oxidized species dihydrobiopterin (BH2) and biopterin (B), remained virtually undetectable in stimulated *Gch1*^*fl/fl*^Tie2cre macrophages, despite a significant upregulation of biopterins in wild-type macrophages (Fig. 2C). To determine whether the biopterin salvage pathway was intact we stimulated BH4 production by supplementation with sepiapterin, which augments BH4 levels via the salvage pathway, independent of de novo BH4 biosynthesis by GTPCH. Sepiapterin supplementation significantly increased intracellular BH4 in the *Gch1*^fl/fl^Tie2cre cells, restoring them to wild-type levels (Fig.2D). This shows that iNOS enzymatic activity is otherwise unaffected and the effects seen are due to a lack of cofactor availability in the *Gch1*^fl/fl^Tie2cre cells.

**Fig. 3:**
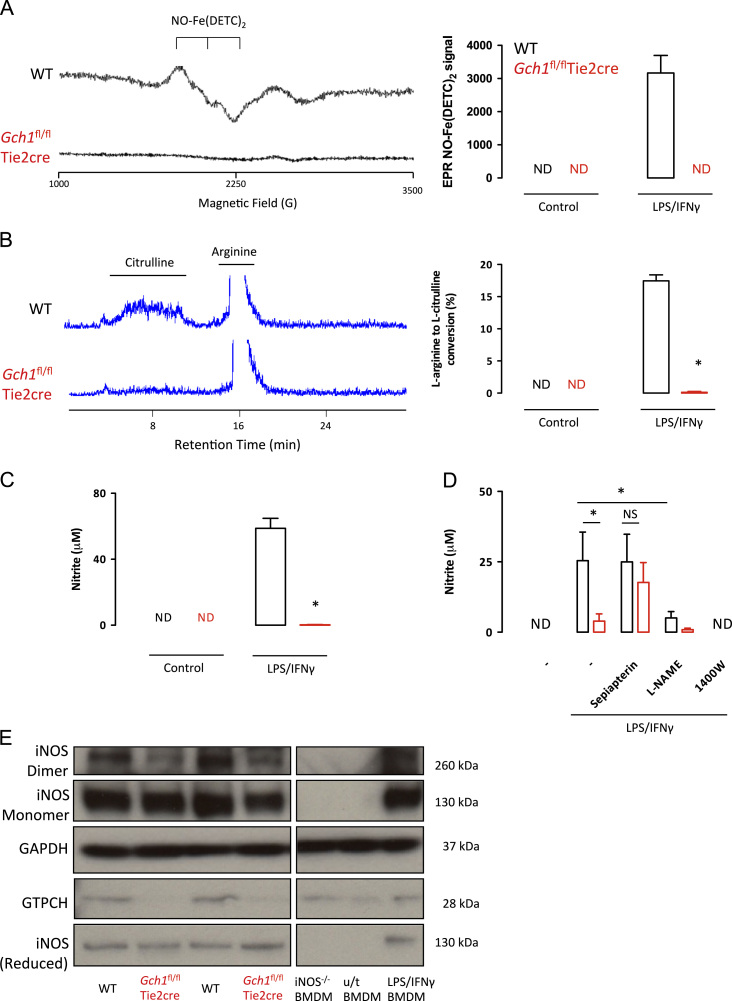
(A) Representative EPR spectra from wild-type (WT) and *Gch1*^fl/fl^Tie2cre macrophages following activation with LPS (100 ng/ml) and IFNγ (10 ng/ml). The characteristic triple-peaked trace associated with NO-Fe(DETC)_2_ signal was readily detectable in the wild-type cells, but absent in the *Gch1*^fl/fl^Tie2cre cells. Quantification of the EPR signal indicated nitric oxide production only by activated wild-type cells (*n*=3). (B) Representative HPLC traces showing arginine to citrulline conversion by wild-type macrophages, which was absent in *Gch1*^fl/fl^Tie2cre cells. Quantification of the NMA-inhibitable arginine to citrulline conversion indicated significantly reduced NOS enzyme activity in the *Gch1*^fl/fl^Tie2cre macrophages. (C) Nitrite accumulation in the cell culture media over 24 h of cell stimulation was measured using the Griess assay. No nitrite production was detected in the absence of stimulation with LPS/IFNγ, but after stimulation was readily detected in wild-type supernatants, but was virtually undetectable levels in the *Gch1*^fl/fl^Tie2cre cells (*n*=6, *P*<0.05). (D) The production of nitrite was significantly reduced by treatment with L-NAME (2 mM) over the 24 h stimulation in wild-type cells, and was completely blocked by incubation with iNOS inhibitor 1400W (10 nM). Production of nitrite by *Gch1*^fl/fl^Tie2cre cells was significantly enhanced by coincubation of the cells with 5 μM sepiapterin during the 24 h cell stimulation. (E) Macrophage lysates from activated cells were run under nonreducing conditions to assess iNOS monomer and dimer formation. Total iNOS and GTPCH content was assessed from duplicate reduced lysate samples under standard blotting conditions. The identity of the bands obtained were confirmed using control lysates from wild-type nonactivated (u/t BMDM) and activated macrophage lysates (LPS/IFNγ BMDM) and activated iNOS^*−*/*−*^ lysates (iNOS^*−*/*−*^ BMDM).

We next determined the effects of macrophage *Gch1* deletion and BH4 deficiency on NO generation, using three different methods. First, we measured authentic NO generation from macrophages using EPR detection of NO using the spin-trap colloid Fe(DETC)_2_. The characteristic NO-Fe(DETC) _2_ EPR triplet was readily demonstrated in activated wild-type macrophages, but was not detectable in *Gch1*^*fl/fl*^Tie2cre macrophages (Fig. 3A). A similar result was observed using radiolabeled L-arginine to L-citrulline conversion using HPLC with on-line scintillation detection. No L-citrulline production was detected in *Gch1*^*fl/fl*^Tie2cre macrophages, compared with more than 15% conversion of L-arginine to L-citrulline in activated wild-type cells (Fig. 3B). Finally, we measured nitrite production by macrophages using the Greiss assay. Wild-type macrophages accumulated high levels of nitrite (>50 µM) in culture medium 24 h after stimulation with LPS and interferon-γ, whereas nitrite was barely detectable in medium from *Gch1*^*fl/fl*^Tie2cre macrophages (Fig. 3C). To test the specificity of the nitric oxide production the cells were treated with either the nonselective NOS inhibitor L-NAME or the iNOS selective inhibitor 1400 W (Fig. 3D). The accumulation of nitrite was significantly reduced in wild-type cells by both inhibitors. To confirm whether the lack of nitric oxide production by the *Gch1*^fl/fl^Tie2cre cells was purely due to a lack of BH4, *Gch1*^fl/fl^Tie2cre macrophages were activated in the presence of sepiapterin, to restore BH4 levels by the salvage pathway, which restored nitrite levels to wild-type levels (Fig. 3D). To further assess alterations to iNOS activity in the absence of BH4 we performed Western blotting of the native protein to determine any contribution of BH4 to the dimerization of iNOS. We found a decrease in dimeric iNOS in the *Gch1*^fl/fl^Tie2cre macrophages. In both wild-type and *Gch1*^fl/fl^Tie2cre cells a prominent signal for monomeric iNOS was detected (Fig. 3E). A parallel aliquot of the lysate was prepared under normal reducing conditions to confirm that the total iNOS expression was unchanged between genotypes. Taken together, these observations demonstrate that macrophage *Gch1* deletion renders iNOS inactive for NO generation that is mediated by BH4 deficiency.

**Fig. 4:**
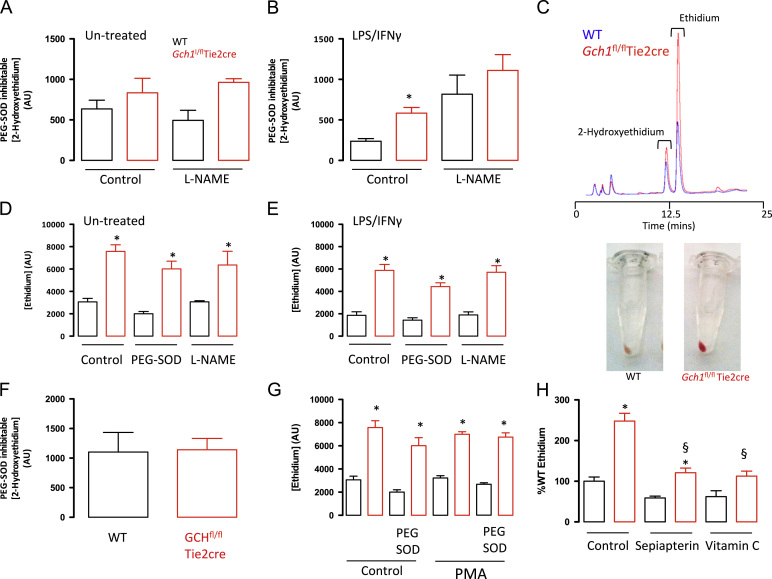
Following activation with LPS (100 ng/ml) and IFNγ (10 ng/ml) cells or control cells were pretreated with or without PEG-SOD (100 U/ml) for 30 min prior to incubation with dihydroethidium for 30 min. The production of 2-hydroxyethidium and ethidium was quantified using HPLC with fluorescence detection. The specificity of the 2-hydroxyethidium signal to superoxide was confirmed by quantification of the PEG-SOD-inhibitable signal. (A) In the absence of cell stimulation no significant difference in superoxide was detected between wild-type (WT) and *Gch1*^fl/fl^Tie2cre cells. (B) Following activation *Gch1*^fl/fl^Tie2cre cells produced significantly more superoxide than wild-type cells. The superoxide signals detected were not inhibitable by L-NAME (2 mM). (C) Representative HPLC trace showing the alterations in dihydroethidium oxidation in wild-type and *Gch1*^fl/fl^Tie2cre cells. Notably *Gch1*^fl/fl^Tie2cre cell pellets were bright red in color (ethidium) following the experiment. (D) Quantification of the ethidium signal indicated a significant increase in *Gch1*^fl/fl^Tie2cre cells under both basal and stimulated conditions (E). The elevated ethidium signal was not significantly inhibited by PEG-SOD (100 U/ml) or L-NAME (2 mM). (F) Superoxide burst was induced by treatment with 2 μM PMA in the presence of dihydroethidium. The PEG-SOD-inhibitable production of superoxide was not significantly difference between genotypes. (G) Induction of the superoxide burst did not alter the elevated production of ethidium by the *Gch1*^fl/fl^Tie2cre cells. (H) Pretreatment of the cells with Sepiapterin for 24 h or vitamin C for 30 min significantly reduced the elevated ethidium signal detected in the *Gch1*^fl/fl^Tie2cre cells (*n*=3–6 per group, *P*<0.05). *P<0.05, difference by genotype; § *P*<0.05, difference from control. Two-way ANOVA.

To investigate the effects of *Gch1* deletion and BH4 deficiency on macrophage ROS production, we next measured O_2_^•*−*^ and other ROS in bone marrow-derived macrophages by quantification of 2-hydroxyethidium (2-HE) and ethidium (E) production from dihydroethidine, using HPLC. The production of 2-HE was significantly elevated in macrophages from *Gch1*^*fl/fl*^Tie2cre mice compared with wild-type cells, but only in the LPS and interferon-γ-activated macrophages (Fig. 4A–C). The specificity of the production of 2-HE was confirmed by analyzing the PEG-SOD-inhibitable signal. While a clear production of superoxide is detectable in both the stimulated and the unstimulated macrophages, this production is not significantly altered by coincubation with L-NAME. In the case of the ethidium signal, the production is significantly increased in the *Gch1*^*fl/fl*^Tie2cre macrophages under both unstimulated and activated conditions (Fig. 4D and E). This overproduction of ethidium by the *Gch1*^*fl/fl*^Tie2cre cells was particularly striking, being visible as an alteration in the color of the cell pellets following dihydroethidium incubation (Fig. 4C). This signal was not inhibited by coincubation with PEG-SOD, nor with the NOS inhibitor, L-NAME (2 mM), which produced little effect. These data indicate a role for BH4 in regulating both superoxide production in the presence of iNOS expression and in the production of other ROS species that are independent of NOS expression. Having observed significant alterations in the production of multiple ROS species in the BH4-deficient cells, we next assessed how the cells would react to a potent superoxide signal, induction of the oxidative burst by PMA. The magnitude of the superoxide signal was not significantly different between genotypes (Fig. 4F). In addition, the production of this large superoxide signal did not alter the detected ethidium signal (Fig. 4G), indicating that the ethidium signal is independent of superoxide production by the cells. To further probe the origin of the elevated ethidium signal, cells were subject to either rescue of intracellular BH4 levels, by addition of sepiapterin, or treatment with a nonspecific antioxidant, Vitamin C (Fig. 4H). In both cases the ethidium signal was significantly decreased in the *Gch1*^*fl/fl*^Tie2cre macrophages, indicating that the increased ethidium signal is the result of intracellular oxidants. These experiments indicate that ROS levels within the cells are altered in a BH4-dependent manner both in the presence and in the absence of iNOS protein expression.

### Macrophage BH4 deficiency and loss of NO lead to altered inflammatory responses and gene expression

**Fig. 5:**
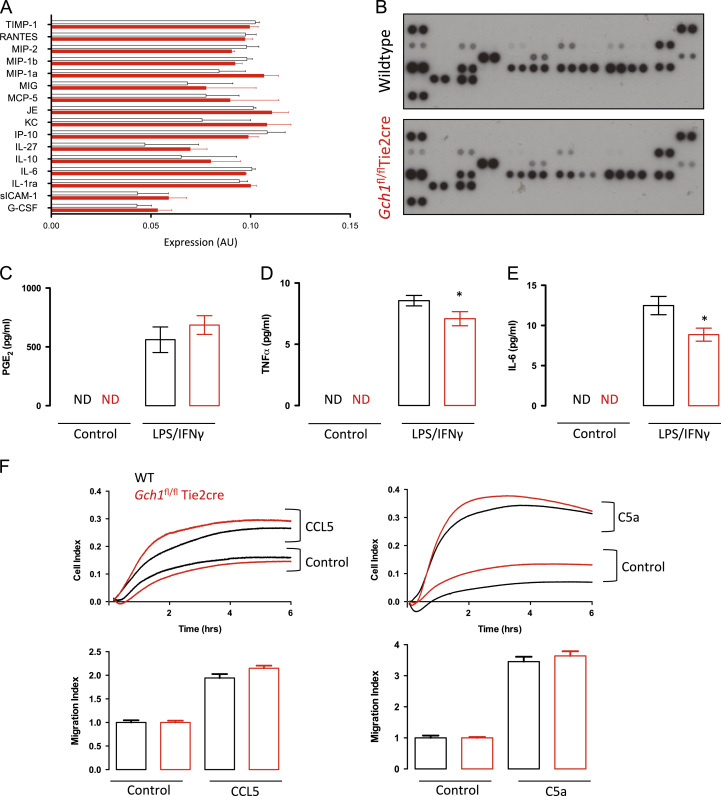
(A) Supernatants were collected from wild-type (WT) and *Gch1*^fl/fl^Tie2cre macrophages 24 h after stimulation with LPS (100 ng/ml) and IFNγ (10 ng/ml). Proteome Profiler arrays, cytokine detection antibodies prespotted onto nitrocellulose filters in duplicate, were used to profile cytokine secretion. The intensity of the cytokine signal was quantified using densitometry for *n*=4 biological replicates per genotype. (B) Representative cytokine arrays show no significant differences between genotypes. (C) Detection of PGE2 by enzyme immunoassay indicated no alteration in production by *Gch1*^fl/fl^Tie2cre cells. (D) Detection of TNFα and IL-6 (E) by ELISA indicated induction of both cytokines by stimulation, with a small but significant reduction in cytokine production by the *Gch1*^fl/fl^Tie2cre cells (cytokine detection *n*=6 per genotype, *P*<0.05). (E) Representative trace from the RTCA-DP software showing macrophage migration toward chemoattractant or buffer control. Quantification of the response in the xCELLigence assay showed that migration toward either CCL5 or C5a was not significantly altered in *Gch1*^fl/fl^Tie2cre macrophages (*n*=3 per genotype).

Next we investigated the requirement for macrophage BH4 synthesis in the inflammatory response to LPS and interferon-γstimulation. A systematic quantification of the secretion of a range of cytokines and chemokines, using a protein array, revealed that the production of mediators including MIP-2, MIP1α , MIG, MCP-5, IL-27, IL-10, IL-6, sICAM-1, G-CS,F and TIMP-1 was not significantly different between wild-type and *Gch1*^*fl/fl*^Tie2cre macrophages (Fig. 5A and B). We also measured cytokines induced by proinflammatory stimulation in macrophage culture media by ELISA, revealing a small but statistically significant reduction in TNFα and IL-6 secretion by *Gch1*^*fl/fl*^Tie2cre macrophages (Fig. 5C and D). More surprisingly, secretion of PGE2 was not affected, as reduced PGE2 production is reported by iNOS^*−*/*−*^ macrophages (Fig. 5E) [26]. To further investigate the effects of *Gch1* deletion on macrophage function, chemotaxis assays were performed to assess cell motility. The BMDMs showed significant migration in response to both classical chemokines (CCL5) and chemoattractants (C5a) (Fig. 5F). However, there was no significant difference in cell migration between wild-type and *Gch1*^*fl/fl*^Tie2cre macrophages (Fig. 5F). Taken together, these data reveal a selective impact of BH4 deficiency on cell redox status, with alterations in both nitric oxide and ROS production, in the absence of large changes in other cell functions.

**Fig. 6:**
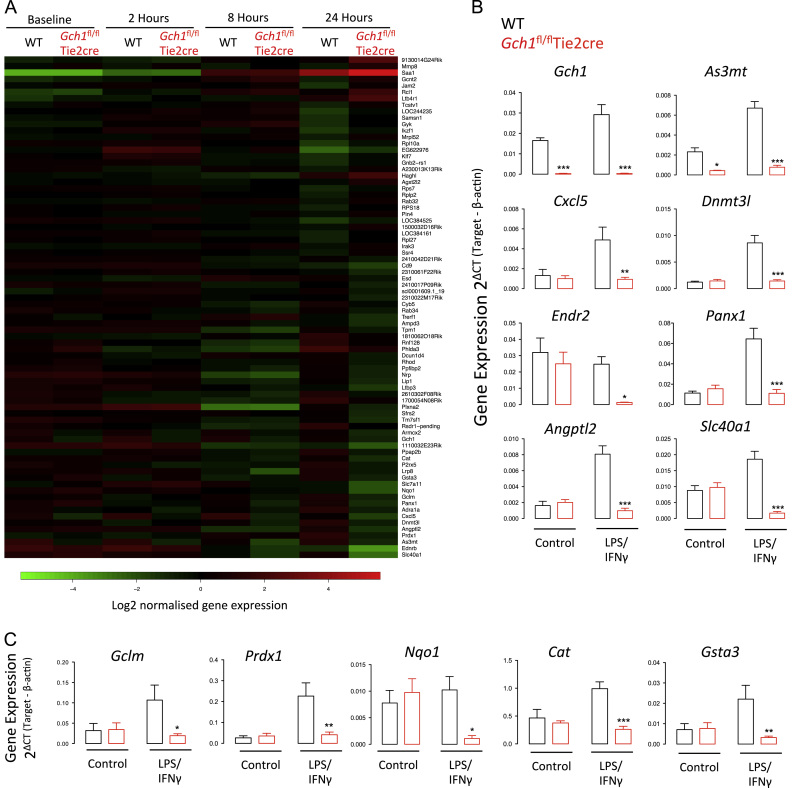
(A) Heat map showing gene expression of genes significantly regulated by >1.5-fold passing a fdr *P*<0.05 across a time course of gene expression. All genes significantly regulated by genotype in at least one time point are shown, ranked by fold difference between wild-type (WT) and *Gch1*^fl/fl^Tie2cre macrophages at 24 h (*n*=3 per genotype per time point, paired data). Data shown are the average normalized log2 expression value for each gene normalized to the expression across all samples. Green represents a positive change and red represents a negative change. (B) Confirmation of gene array result by quantitative RT-PCR of RNA from cells with or without stimulation with LPS (100 ng/ml) and IFNγ (10 ng/ml) for 24 h. (C) Confirmation of decreased expression of NRF2 target genes 24 h following stimulation as above. (RT-PCR studies *n*=6 per genotype, two-way ANOVA *P<0.05, ** *P*<0.01, ***P<0.001 for difference by genotype).

We next investigated how the loss of NO production and increased ROS production induced by loss of BH4 would alter gene expression in macrophages after LPS and interferon-γ stimulation, using gene array analysis of macrophage mRNA (Fig. 6A). Macrophages from wild-type and *Gch1*^*fl/fl*^Tie2cre mice were harvested at baseline or 2, 8, or 24 h after stimulation with LPS and interferon-γ. The change in gene expression in response to inflammatory stimulation was similar between genotypes, reflecting the broadly similar levels of cytokine and chemokine secretion. Pairwise comparison of gene expression between wild-type and *Gch1*^fl/fl^Tie2cre macrophages at baseline revealed the expected decrease in *Gch1* gene expression and in addition a significant reduction in arsenite-3-methlytransferase (*As3mt)* expression (Supplementary Table 1). This minimal *Gch1*-dependent change in gene expression was also seen at 2 h post stimulation. At 8 h after stimulation a significant decrease in *Cxcl5* expression was seen in addition to the changes in *As3mt* expression (Supplementary Table 1). However, 24 h after stimulation 78 genes were altered in *Gch1*^fl/fl^Tie2cre cells (Fig. 6A and Supplementary Table 1). Validation of selected genes significantly altered in *Gch1*^fl/fl^Tie2cre cells, by RT-PCR, confirmed significant upregulation of genes in wild-type macrophages that were strikingly reduced or absent in *Gch1*^*fl/fl*^Tie2cre macrophages (Fig. 6B). This validation was performed both with the RNA samples used for the gene array and with further RNA samples from additional biological replicates.

The gene array revealed changes in genes related to the BH4-dependent enzyme pathways of catecholamine synthesis, α-1-adrenoceptor, and the AGMO-dependent ether lipid metabolism pathway, platelet activating factor acetyl hydrolase (*Pafah*) (Supplementary Table 1). Ingenuity Pathway analysis of the genes significantly altered at 24 h revealed a significant modulation of the NRF2 pathway, predicting a decrease in upstream NRF2 activity in the *Gch1*^fl/fl^Tie2cre cells. Pathway analysis predicts decreased activation of NRF2 following reduced expression of 6 out 8 key NRF2 targets in *Gch1*^fl/fl^Tie2cre cells (*P*<0.05). The identification of altered NRF2 target gene expression was confirmed by RT-PCR of analysis of established NRF2-dependent genes (*Gclm*, *Prdx1*, *Gsta3*, *Nqo1*, and *Catalase*) showing an apparent decrease in NRF2 activity in *Gch1*^fl/fl^Tie2cre macrophages (Fig. 6C).

## Discussion

We have created a novel mouse model of *Gch1* deletion in order to test the specific requirements for BH4 in macrophage redox biology. This novel mouse model reveals several new and important findings. First, *Gch1* deletion leads to near total macrophage BH4 deficiency, which in turn leads to loss of iNOS-derived NO bioactivity. Second, macrophage ROS production is increased by BH4 deficiency and is in part iNOS mediated but is also observed in the absence of iNOS expression. Third, analysis of macrophage gene expression reveals specific alteration to a set of 78 genes following iNOS induction that are dependent on BH4 levels. In particular, NRF2-dependent genes showed reduced induction in *Gch1*^fl/fl^Tie2cre macrophages. Taken together, these data reveal a requirement for BH4 in regulating cellular redox state, through direct effects on iNOS-mediated NO, ROS production and through downstream alterations in NRF2-dependent gene expression.

The identification of these specific requirements for *Gch1*, BH4, and iNOS-dependent NO generation has important implications for understanding the regulation of macrophage activation and gene expression. NO has been reported to have multiple divergent effects on cytokine production, with both induction and inhibition of cytokine production reported following treatment with NO donors [27]. However, our data now show that macrophage cytokine production following inflammatory stimulation is largely preserved even when iNOS-dependent NO production is absent. Conversely, the specific reduction in NRF2-dependent gene expression in BH4-deficient macrophages strongly supports a requirement for NO in activation of NRF2 by nitrosylation of Keap1 [28], even though NRF2 can also be induced by ROS [4]. In this regard, the observations in *Gch1*-deficient macrophages are concordant with those from *Nos2*^*−*/*−*^ mice that show decreased activation of NRF2 in response to proinflammatory stimuli [29]. However, given the large alterations in NO-ROS balance in the *Gch1*^fl/fl^Tie2cre macrophages we cannot fully rule out ROS-mediated alterations in NRF2 activation. In contrast, gene expression analysis in BH4-deficient macrophages following activation identifies a novel subset of genes that are dependent on iNOS, but not on NO production, thus implicating other iNOS signaling roles such as ROS production. Dissecting out the relative roles of iNOS-derived NO vs other iNOS effects cannot be determined using the *Nos2*^*−*/*−*^ mouse, where all iNOS effects will be absent. For example, *Nos2*^*−*/*−*^ macrophages are reported to have a striking lack of PGE2 induction, hypothesized to be via reduced S-nitrosylation of COX2. However, we observed no significant alteration in PGE2 levels in BH4-deficient macrophages, indicating that other roles for iNOS may mediate this activation of COX2 [26].

The potential importance of BH4 in regulating ROS-mediated effects in macrophages is supported by striking increases in superoxide production in BH4-deficient activated macrophages that parallels loss of NO generation by iNOS. This novel system allows for the first time a comparison between NO-mediated and ROS-mediated effects of iNOS induction in macrophages. While the production of superoxide by iNOS has been reported, the precise control of ROS production by BH4 is more controversial. Some studies have shown that iNOS becomes uncoupled by a lack of BH4 and that superoxide-induced oxidative loss of BH4 causes ROS formation [30]. Restriction of arginine availability is also reported to cause increased ROS production by iNOS [31], resulting in peroxynitrite formation [32]. Recent studies have shown that the ferrous inducible oxygenase domain of iNOS remains monomeric in the absence of BH4 and produces superoxide [33]. However, isolated protein systems have shown that the formation of the iNOS dimer, which is required for NO production, is more BH4 dependent than other NOS isoforms [8]. In whole cell and body systems iNOS uncoupling is observed, with reduced BH4 causing increased iNOS uncoupling in T-cells and in renal allografts associated with a BH4-inhibitable superoxide production [34,35]. In addition to enhanced superoxide production, which may be a result of iNOS uncoupling, the more striking effect of loss of BH4 on cellular redox state is the enhanced ethidium signal detected. This signal is independent of NOS expression, indicating for the first time that the physiological level of BH4 has a role in controlling cellular redox state. The source of this ROS signal is unclear; it seems unlikely to be via NOX2 since induction of the phagocytic oxidative burst by PMA is unaltered between genotypes. These data indicate that basal ROS production from other sources may be elevated, or that BH4 forms part of the physiological intracellular redox buffering capacity of the cell.

The ability of macrophages to produce high levels of ROS and RNS requires macrophages themselves to induce cell processes that allow protection from the local aggressive local environment. This protection is mediated in part through induction of redox-sensitive transcription factors, such as HIF and NRF2 [29]. In addition to the core group of NRF2-regulated genes that show altered regulation in the *Gch1*^fl/fl^Tie2cre mice, other gene expression changes reflect changes previously associated with NO production. For example, *Slc40a1* (Ferroportin) gene expression has been reported to be induced by nitric oxide, with a failure of induction in iNOS^*−*/*−*^ mice [36]. However, more interestingly genes that showed altered activation in the *Gch1*^fl/fl^Tie2cre macrophages have not previously been associated with iNOS or nitric oxide biology, suggesting that changes in iNOS-mediated redox signaling, rather than only NO, have important biological effects. Expression of As3MT (arsenic(III)-methyltransferase) was significantly different in BH4-deficient macrophages at all time points in the gene array analysis, irrespective of LPS and IFNγ stimulation, indicating that the alteration in expression is independent of iNOS, but dependent on BH4 and *Gch1*. As3MT mediates arsenic methylation and is involved in the endogenous arsenic detoxification process, with *As3MT*^*−*/*−*^ mice showing slower excretion of arsenic with enhanced symptoms of arsenic toxicity [37]. While this gene has not been previously reported to be altered by ROS or RNS, it is perhaps telling that expression of this protein is associated with alteration in the redox status of cells, with its activity being increased by glutathione [38]. These data implicate a potential general role for BH4 in arsenic metabolism, which warrants investigation of this process in more relevant cell types. The function of As3MT in the macrophages studied here, in the absence of arsenic, is unclear. Other genes altered in the *Gch1*^fl/fl^Tie2cre cells have potent roles in cellular function; *Cxcl5* encodes a chemokine that can recruit both neutrophils and monocytes [39]. Dnmt3l protein is a member of the de novo DNA methyltransferases, indicating the possibility of further downstream epigenetic alterations in BH4-deficient cells [40]. Endr2 (Endothelin receptor 2) and Angptl2 (Angiopoietin-like protein 2) have both been highlighted to play roles in vascular inflammation and Panx1 (Pannexin 1) has been linked to the 5-lipoxygenase pathways in murine macrophages [41–43].

In summary, we describe for the first time that deficiency of BH4 in macrophages, as a result of targeted *Gch1* deletion, prevents nitric oxide formation by iNOS, but maintains or increases ROS production, allowing for the first time a comparison of NO vs ROS-mediated effects of iNOS in macrophage inflammatory signaling. Multiple effects of *Gch1* deletion on cellular ROS levels are seen both in the presence and in the absence of iNOS expression. These changes in macrophage redox state are not sufficient to cause widespread changes in the macrophage inflammatory state or cellular migration. However loss of iNOS-mediated NO production leads to specific alterations in the expression of NRF2 target genes in the absence of BH4, along with further BH4-dependent iNOS-independent changes in gene expression. Identification of BH4-mediated NO vs ROS effects in macrophage inflammatory responses highlights new complexity in iNOS biology that has not been hitherto addressed by models of iNOS deletion, and have important implications for understanding the role of iNOS, NO, and BH4 in inflammation, immunity, and host defense.
