# Supplementary material for: Regulation of iNOS function and cellular redox state by macrophage Gch1 reveals specific requirements for tetrahydrobiopterin in NRF2 activation
Source: Free Radic Biol Med. 2015 Feb;79:206–16. doi: 10.1016/j.freeradbiomed.2014.10.575 (PMC4344222; doi:10.1016/j.freeradbiomed.2014.10.575)
Supplement: Supplementary file 1 — Supplementary Material [file mmc1.pdf]

Timepoint	Gene Symbol	Gene Name	Fold Change KO/WT (log 2)	adj.P.Val (fdr<0.05)
**Baseline**				
	**Gch1**	**GTP cyclohydrolase 1**	**-2.504137152**	**0.007522071**
	**As3mt**	**arsenic (+3 oxidation state) methyltransferase**	**-1.961859611**	**0.01081917**
	2010002M12Rik	IKEN cDNA 2010002M12 gene	1.587273677	0.042230945
**2 hours**				
	**Gch1**	**GTP cyclohydrolase 1**	**-1.765541701**	**0.001665967**
	**As3mt**	**arsenic (+3 oxidation state) methyltransferase**	**-2.118929192**	**0.003958463**
**8 hours**				
	**As3mt**	**arsenic (+3 oxidation state) methyltransferase**	**-2.332543041**	**0.001186035**
	**Gch1**	**GTP cyclohydrolase 1**	**-2.701844945**	**0.001186035**
	**Cxcl5**	**chemokine (C-X-C motif) ligand 5**	**-2.355100886**	**0.011271599**
**24 hours**				
	9130014G24Rik	Androglobin	2.272164489	0.029270611
	Mmp8	Matrix Metallopeptidase 8	1.744466193	0.020730612
	Saa1	Serum amyloid A1	1.614255153	0.031037374
	Gcnt2	glucosaminyl (N-acetyl) transferase 2	1.571481215	0.022405328
	Jam2	junction adhesion molecule 2	1.558280674	0.021063142
	Rcl1	RNA terminal phosphate cyclase-like 1	1.48393848	0.021155879
	Ltb4r1	leukotriene B4 receptor 1	1.357425337	0.0488061
	Tcstv1	2-cell-stage, variable group, member 1	1.334797055	0.04709696
	LOC244235	similar to 3110003A17Rik protein	1.325647788	0.010416919
	Samsn1	SAM domain, SH3 domain and nuclear localization signals 1	1.284696018	0.028231789
	Gyk	glycerol kinase	1.273778797	0.0488061
	Ikzf1	IKAROS family zinc finger 1	1.255774782	0.046932192
	Mrpl52	mitochondrial ribosomal protein L52	1.199359854	0.013644648
	Rpl10a	ribosomal protein L10a	1.192567643	0.021063142
	EG622976	predicted gene 6377	1.191636873	0.04822353
	Klf7	Kruppel-like factor 7	1.154753082	0.011107966
	Gnb2-rs1	guanine nucleotide binding protein (G protein), beta polypeptide 2-like 1	1.126250054	0.020730612
	A230013K13Rik	Unknown	1.086764555	0.021063142
	Haghl	hydroxyacylglutathione hydrolase-like	1.015728151	0.012803124
	Agxt2l2	alanine-glyoxylate aminotransferase 2-like 2	0.980404235	0.021155879
	Rps7	ribosomal protein S7	0.927150453	0.029270611
	Rplp2	ribosomal protein, large, P2	0.922427699	0.036444038
	Rab32	RAB32, member RAS oncogene family	0.853098726	0.046932192
	RPS18	ribosomal protein S18	0.84872402	0.020730612
	Pin4	protein (peptidylprolyl cis/trans isomerase) NIMA-interacting, 4	0.845908082	0.029771758
	LOC384525	predicted gene 5321	0.834892928	0.046264379
	1500032D16Rik	NADH dehydrogenase (ubiquinone) flavoprotein 3	0.810407504	0.02720835
	LOC384161	similar to 60S ribosomal protein L11	0.799766949	0.029270611
	Rpl27	ribosomal protein L27	0.780928591	0.04590193
	Irak3	interleukin-1 receptor-associated kinase 3	0.770832632	0.047540493
	Ssr4	signal sequence receptor, delta	0.763446064	0.046445081
	2410042D21Rik	katanin p80 subunit B like 1	-0.779217626	0.031037374
	Cd9	CD9 molecule	-0.846804029	0.043073275
	2310061F22Rik	cytosolic thiouridylase subunit 2 homolog	-0.875696642	0.029270611
	2410017P09Rik	peptidyl-tRNA hydrolase domain containing 1	-0.897902021	0.031037374
	scl0001609.1_19	unknown	-0.92411087	0.02277731
	2310022M17Rik	biorientation of chromosomes in cell division 1	-0.931825438	0.033409208
	Cyb5	cytochrome b-5	-0.973928128	0.021155879
	Rab34	RAB34, member RAS oncogene family	-0.978624285	0.031037374
	Trerf1	transcriptional regulating factor 1	-1.040293167	0.048526764
	Ampd3	adenosine monophosphate deaminase 3	-1.052141763	0.028231789
	Tpm1	tropomyosin 1	-1.053932142	0.021063142
	1810062O18Rik	RIKEN cDNA 1810062O18 gene	-1.067032815	0.029270611
	Rnf128	ring finger protein 128	-1.074718892	0.043237162
	Phlda3	pleckstrin homology-like domain, family A, member 3	-1.088304873	0.034290234
	Dcun1d4	DCN1, defective in cullin neddylation 1, domain containing 4	-1.109155147	0.029270611
	Rhod	ras homolog family member D	-1.126055054	0.031037374
	Esd	esterase D	-1.143809037	0.021155879
	Nrp	neural regeneration protein	-1.195503189	0.031167202
	Lip1	sphingosine N-acyltransferase subunit LIP1	-1.212327709	0.021452491
	Ltbp3	latent transforming growth factor beta binding protein 3	-1.218656026	0.021063142
	2610302F08Rik	Unknown	-1.236840512	0.034290234
	1700054N08Rik	centriole, cilia and spindle associated protein	-1.238743247	0.04590193
	Plxna2	plexin A2	-1.257114382	0.04590193
	Sfrs2	splicing factor arginine/serine rich 2	-1.261649406	0.010044201
	Ppfibp2	PTPRF interacting protein, binding protein 2	-1.262026296	0.01150181
	Tm7sf1	G protein-coupled receptor 137B	-1.280086867	0.042743788
	Rsdr1	dehydrogenase/reductase (SDR family) member 3	-1.305996643	0.036444038
	Armcx2	armadillo repeat containing, X-linked 2	-1.361596907	0.021510636
	1110032E23Rik	family with sequence similarity 198, member B	-1.503006992	0.02277731
	Ppap2b	phosphatidic acid phosphatase type 2B	-1.585431469	0.011107966
	**Cat**	**catalase**	**-1.605430534**	**0.016416791**
	P2rx5	purinergic receptor P2X, ligand-gated ion channel, 5	-1.624698879	0.02277731
	Lrp8	low density lipoprotein receptor-related protein 8	-1.627868865	0.023197322
	**Gsta3**	**glutathione S-transferase alpha 3**	**-1.679723838**	**0.046264379**
	Slc7a11	solute carrier family 7	-1.692881843	0.020730612
	**Nqo1**	**NAD(P)H dehydrogenase, quinone 1**	**-1.729702014**	**0.012803124**
	**Gch1**	**GTP cyclohydrolase 1**	**-1.754774124**	**0.021510636**
	**Gclm**	**glutamate-cysteine ligase, modifier subunit**	**-1.801779329**	**0.046264379**
	Adra1a	adrenoceptor alpha 1A	-1.90142389	0.021155879
	**Cxcl5**	**chemokine (C-X-C motif) ligand 5**	**-1.914496135**	**0.021063142**
	**Dnmt3l**	**DNA (cytosine-5-)-methyltransferase 3-like**	**-1.956920456**	**0.010044201**
	**Panx1**	**pannexin 1**	**-1.980322253**	**0.021155879**
	**Angptl2**	**angiopoietin-like 2**	**-2.010245097**	**0.009778118**
	**Prdx1**	**peroxiredoxin 1**	**-2.014804226**	**0.020730612**
	**As3mt**	**arsenic (+3 oxidation state) methyltransferase**	**-2.442596664**	**0.000222016**
	**Ednrb**	**endothelin receptor type B**	**-2.816621127**	**6.35076E-08**
	**Slc40a1**	**solute carrier family 40**	**-3.249830473**	**2.63466E-05**

Genes confirmed by quantitative RT-PCR

Supplementary Table 1: Gene expression using a whole mouse genome array (Illumina Mouse WG6(v2) Beadchip Array). A total of 24 mouse samples from two experimental groups, over 4 timepoints: WT and Gch1fl/flTie2cre at baseline, 2, 8 or 24 hours post stimulation (n = 3 per group). Chips were scanned with Illumina BeadArray Reader and GenomeStudio V2010.1 (Illumina Inc) was used for data extraction. Data underwent a variance-stabilisation and normalisation (VSN) algorithm using Limma (24). Normalised data was imported to Bioconductor (R version 2.14) (Biobase) and comparison by genotype were performed for each timepoint to identify significantly differentially expressed genes passing a false discovery rate of <0.05 (25). Genes significantly regulated by >1.5 fold by genotype at each time point, ranked by fold difference between wildtype (WT) and $Gch1^{fl/fl}$Tie2cre macrophages (n=3 per genotype per timepoint, paired data) are shown. Data is the (log2) fold change between WT and $Gch1^{fl/fl}$Tie2cre and the adj. P value.
